# Death is not always a failure: outcomes from implementing an online virtual patient clinical case in palliative care for family medicine clerkship

**DOI:** 10.3402/meo.v18i0.22711

**Published:** 2013-11-22

**Authors:** Amy Tan, Shelley Paige Ross, Kimberley Duerksen

**Affiliations:** Department of Family Medicine, Faculty of Medicine & Dentistry, University of Alberta, Edmonton, AB, Canada

**Keywords:** palliative care, virtual patient, online learning, end-of-life care, end-of-life decision-making, undergraduate medical education

## Abstract

**Background:**

The dying patient is a reality of medicine. Medical students, however, feel unprepared to effectively manage the complex end-of-life (EOL) management issues of the dying patient and want increased experiential learning in Palliative Care.

**Aims:**

To address the need for more formal curriculum in EOL care, we developed and implemented an online virtual patient (VP) clinical case in Palliative Care into the 2010–2011 Year Three Family Medicine Clerkship rotation curriculum.

**Methods:**

A mixed-method design was used to measure the change in knowledge and perceived preparedness level in EOL care before and after completing the online VP case. A survey collected qualitative descriptions of the students’ educational experience of using this case.

**Results:**

Ninety five percent (130/137) of the students voluntarily consented to have their results analyzed. The group knowledge score (*n*=127) increased significantly from a pre-course average of 7.69/16±2.27, to a post-course average of 10.02/16±2.39 (*p*<0.001). The students’ self-assessed comfort level increased significantly with all aspects of EOL management from pre-course to post-course (*p*<0.001). Nearly, 91.1% of the students rated the VP realism as ‘Good to Excellent’, 86% rated the case as educationally beneficial. Nearly 59.3% of students felt emotionally engaged with the VP. Qualitative feedback found that the case content was very useful and realistic, but that the interface was sometimes awkward to navigate.

**Conclusions:**

The online VP case in Palliative Care is a useful teaching tool that may help to address the need for increased formal Palliative Care experience in medical school training programs.

## Introduction

The dying patient is a reality of medicine. In Canada, the United States, and the United Kingdom, the proportion of seniors is expected to increase from 14% of the population, to 25% by 2036 (1–3). Thus, regardless of discipline, physicians increasingly will require the knowledge, skills, and experience to manage the dying patient in practice.

Studies have shown that North American medical students feel unprepared to provide end-of-life (EOL) care, and desire more experiential learning in Palliative Medicine (4–8). Similarly, Weber et al. (9) surveyed graduating students at two German universities and found that that the majority of these students felt unprepared to provide EOL care. Residents also feel ill-prepared with EOL decision making (10). Canada has recognized the need for palliative care training across the education spectrum (11). The Educating Future Physicians in Palliative and End-of-Life Care (EFPPEC) project aimed to ensure that all medical students and post-graduate trainees receive education in EOL care (11). Despite this, Canadian medical school graduates still feel underprepared: a survey conducted by the Association of American Medical Colleges showed that in 2008, 16.3% of all Canadian graduates felt unprepared in palliative and EOL care; in 2011, 22% of all graduates felt unprepared (12).

To address the need for more formal curriculum in EOL care during medical school at our university, we studied the outcomes of implementing an online virtual patient (VP) clinical case in Palliative Care during the 2010–2011 Family Medicine Clerkship core rotation. A VP case is defined as an ‘interactive computer simulation of real-life clinical scenarios for the purpose of health professionals training, education or assessment’ (13). In a VP case, students are presented with various disease scenarios at an accelerated pace and are able to explore the consequences of their decisions in a more life-like simulation (14, 15). Furthermore, the online VP provides a ‘safe’ environment to explore the potentially emotionally charged issues in EOL care (16). The VP case utilized (17) was developed as part of the Canadian Undergraduate Family Medicine Education Director's (CUFMED) open-access *Canadian Shared Family Medicine Undergraduate Curriculum* (SHARC-FM) (18).

The VP, ‘Emil’, is a 68 year-old man with an 8-month history of non-small cell lung cancer presenting with new onset back pain (17). The ‘*chart your own adventure*’ style format, with short answer and multiple choice questions embedded throughout, follows a 4 to 6 month period from new bony metastases through to hospice admission and death (17). Students experience a simulation of longitudinal care of a terminally ill patient and manage issues from symptom management to psychosocial support for the dying patient and family. Our goal was to determine whether a VP case in palliative care could offer students an acceptable alternative to real-life experiences with EOL care.

## Methods

### Study setting and participants

The VP case was implemented as a mandatory exercise in the 2010–2011 (August–July) Year 3 Family Medicine Clerkship rotation (*n*=137 students) of a 4-year medical program. Ethics approval was attained from our University's Human Research Ethics Board, and voluntary informed consent was obtained to analyze student scores and feedback in an anonymous fashion using unique identifiers.

### Study procedure

A pre-course knowledge test and a level-of-preparedness survey were administered on the first day of the rotation. The pre-course test assessed the students’ knowledge of palliative care, and the survey determined the students’ personal level of preparedness managing various EOL clinical situations (i.e., pain management, symptom control, and discussing limited prognosis status). The students had 8 weeks to review the VP case individually with unlimited access. After the rotation, students were given a post-course test and a survey that included the same knowledge and self-assessment questions as the initial test. Information about the time spent on the VP case and real Palliative Care patient encounters during their rotation was collected. Additionally, general case feedback on each student's experience with the online case, and their input on how the VP could be improved were sought by soliciting written comments. Pre- and post-course tests were matched using unique identifiers.

### Data analysis

Data analysis employed mixed methodology. SPSS 19.0 was used for statistical analysis. Individual and aggregate knowledge pre- and post-course tests/surveys were anonymously compared using a paired *t*-test, as were the self-assessed preparedness ratings. Students’ perception of case realism, educational benefit, and emotional engagement were compared with the time spent on the case, using an independent *t*-test. An *α* level of 0.05 was used to test for statistical significance.

The qualitative feedback data from the student comments underwent thematic analysis with coding. Two of the authors independently coded the data and then discussed to ensure that consensus was reached on the themes and key quotes.

## Results

In the 2010–2011 cohort, 95% (130/137) of the students voluntarily consented to have their results analyzed. Two percent (3/130) could not be analyzed because the unique identifiers could not be matched pre- and post-course.

The average time spent on the VP was 0.93 hours±0.65 hours (range 0–4 hours; *n*=123). The average number of real Palliative Care patient encounters during the 8-weeks rotation was 1.74±2.1 (range 0–15). Nearly, 30.4% of students had no real-life Palliative Care encounters, 25.6% had one encounter and 20.8% had two encounters. The group knowledge score (maximum score of 16) for 127 students increased significantly from a pre-course average of 7.69±2.27, to a post-course average of 10.02±2.39 (*p*<0.001). The students’ self-assessed comfort level increased significantly with all aspects of Palliative Care management when comparing pre- to post-course survey scores (*p*<0.001) (Table 1).

**Table 1:** Student self-reported level of preparedness and experiences following the virtual patient Palliative Care online case

Personal level of preparedness with managing the following palliative care clinical situation		
Pain	Pre: 2.42±0.8 Post: 3.6±0.68	*p*<0.001
Other palliative care symptoms	Pre: 2.21±0.76 Post: 3.26±0.72	*p*<0.001
Counseling patient	Pre: 2.30±0.82 Post: 3.48±0.75	*p*<0.001
Discussing patient's limited prognosis with family	Pre: 2.33± 0.90 Post: 3.43±0.79	*p*<0.001
Discussing the use of a personal directive w/pt.	Pre: 2.16±0.92 Post: 3.28±0.89	*p*<0.001
Taking care of a dying patient in general	Pre: 2.17±0.81 Post: 3.14±0.76	*p*<0.001
All *p*-values from paired *t*-test.		
Thematic analysis of student's narrative comments		

Theme	Comments	

Useful content	‘Found it helpful and realistic, appreciated the in-depth explanations’. ‘It was well written and had a lot of good information in it’.	
Beneficial teaching modality	‘I think it is great way to get us thinking about palliative care. It helped me to learn all of the additional important factors such as family situation, personal directives etc’. ‘It was helpful to study it in a case format so you could understand the steps’.	
Realism of case	‘It was quite good, and taught me a lot. The realism of the case was a plus’. ‘I thought it was a realistic case with good teaching points. A great way to learn the concepts. I wish there were more’.	
Awkward navigation	‘Found the navigation counterproductive & frustrating. I appreciate the attempt to make it more realistic, but I would end up doing it in a very illegal order and I felt like I kept getting interrupted by tangents’.	
Worried about missing key points in the case	‘I don't like the “choose your own adventure” approach. I felt like I was missing parts’. ‘The case was helpful. It would have been nice to have a summary of key points at the end’.	

With regard to the VP case, 91.1% of the students rated the realism of the VP as ‘Good to Excellent’, 86% rated the educational benefit of the case as ‘Good to Excellent’ and 59.3% of students felt emotionally engaged with the VP. Students who spent more than 20 minutes on the VP were significantly more emotionally engaged than those who spent less time (*p*=0.032).

For the qualitative aspect of the post-course survey, 75 consenting students submitted written comments about their experience and provided constructive feedback. Thematic analysis of all qualitative feedback about the VP revealed five key themes. Table 1 summarizes the identified themes and exemplar quotes.

## Discussion

We found that implementing a VP in Palliative Care into the core Year 3 Family Medicine Clerkship curriculum provided beneficial experiential learning. This is important evidence to support virtual cases as viable options to increase medical student exposure to EOL issues and care. The group knowledge test scores increased significantly and students perceived significantly increased comfort levels with EOL management after completing the VP case. Students found the case to be a beneficial and realistic teaching tool. Our findings confirm the work of others with regard to the educational value of using VPs in medical education (19, 20). This study supports the idea that VP cases appear to be widely accepted by students and provide a satisfying learning experience (21, 22).

Student comments revealed that some students struggled with the ‘chart your own adventure’ learning experience and would have preferred a more linear format where they could be ensured that they would not miss any key learning points. Other students, however, found great benefit in the interactive branching case style. These comments may have reflected individual students’ preferred approaches to learning, or individual levels of comfort with the Palliative Care subject matter. While more linear cases do ensure that all learners cover the same material, more exploratory or branching cases allow for increased learner autonomy and a closer simulation to a real-life patient (19).

More experiential learning and positive role modeling of EOL decision-making discussions is required (10) to counteract the troubling dominant message often learned by students that ‘*death is a failure and caring for the dying is not an important part of medicine*’ (23). This VP case is promising as a curricular tool to enhance medical training in EOL management of the dying patient. While there is no consensus as to the most effective method to deliver Palliative Care education (24), the ideal method likely needs to include a variety of modalities, such as Palliative Care clinical rotations, ward-based assignments, ‘death rounds’, online discussions groups, and VPs (8, 11, 25, 26), provided in an integrated fashion to encompass the complexities of Palliative Care. Effective EOL teaching improves students’ attitudes about death and EOL care (25).

### Limitations

This was a study conducted with one cohort of students at one medical school. We could not find a correlation between the individual time spent on the online case and an improvement in their individual knowledge test scores. Interestingly, we also could not find any correlation between the number of real Palliative Care patient encounters a student had and an improvement in their individual knowledge test scores. Perhaps, hearing about the VP case at the rotation's Orientation session heightened the students’ awareness of Palliative Care.

We discovered that it might have been illuminating to elicit specific details of real patient encounters to determine to what extent the students’ real Palliative Care encounters provided hands-on experience. Ascertaining which aspects of EOL were encountered, and what part of the illness trajectory was experienced, would have helped specify how this online VP case supported the Palliative Care clinical experience.

### Future directions

Future work could include expanding the knowledge component of this study in an attempt to better capture changes in students’ knowledge and plans to track the perceived preparedness change over a longer period of time. It is imperative to continue work to determine the best methods to deliver effective clinical Palliative Care learning opportunities to better prepare medical school graduates to provide EOL care to Canada's aging population.

## Conclusions

The online VP case in Palliative Care is a useful teaching tool that may help to address the need for increased formal Palliative Care experience in medical school training programs. The inclusion of many EOL psychosocial management issues and the exploration of the EOL ethics provided in this VP case may also aid students to more confidently manage potentially emotionally charged EOL situations (5). Furthermore, VP cases may be valuable to enhance students’ experiential teaching for other clinical presentations that are not prevalent or would benefit from a longitudinal exposure not always achievable in discrete rotations.
